# Diaqua­bis(2-bromo­benzoato-κ*O*)bis­(nicotinamide-κ*N* ^1^)nickel(II)

**DOI:** 10.1107/S1600536809021710

**Published:** 2009-06-13

**Authors:** Tuncer Hökelek, Filiz Yılmaz, Barış Tercan, F. Elif Özbek, Hacali Necefoğlu

**Affiliations:** a Department of Physics, Hacettepe University, 06800 Beytepe, Ankara, Turkey; b Department of Chemistry, Faculty of Science, Anadolu University, 26470 Yenibağlar, Eskişehir, Turkey; c Department of Physics, Karabük University, 78050 Karabük, Turkey; d Kafkas University, Department of Chemistry, 63100 Kars, Turkey

## Abstract

The title Ni^II^ complex, [Ni(C_7_H_4_BrO_2_)_2_(C_6_H_6_N_2_O)_2_(H_2_O)_2_], is centrosymmetric. It contains two 2-bromo­benzoate (BB) ligands, two nicotinamide (NA) ligands and two water mol­ecules, all of them being monodentate. The four O atoms in the equatorial plane around the Ni atom form a slightly distorted square-planar arrangement, while the slightly distorted octa­hedral coordination is completed by the two N atoms of the NA ligands in the axial positions. The dihedral angle between the carboxyl­ate group and the adjacent benzene ring is 30.81 (17)°, while the pyridine and benzene rings are oriented at a dihedral angle of 84.66 (6)°. In the crystal structure, O—H⋯O and N—H⋯O hydrogen bonds link the mol­ecules into a supra­molecular structure. A weak C—H⋯π inter­action is also found.

## Related literature

For general background, see: Antolini *et al.* (1982 ▶); Bigoli *et al.* (1972 ▶); Krishnamachari (1974 ▶); Nadzhafov *et al.* (1981 ▶); Shnulin *et al.* (1981 ▶). For related structures, see: Hökelek *et al.* (2009*a* ▶,*b* ▶,*c* ▶); Özbek *et al.* (2009 ▶); Sertçelik *et al.* (2009*a* ▶,*b* ▶,*c* ▶); Tercan *et al.* (2009 ▶).
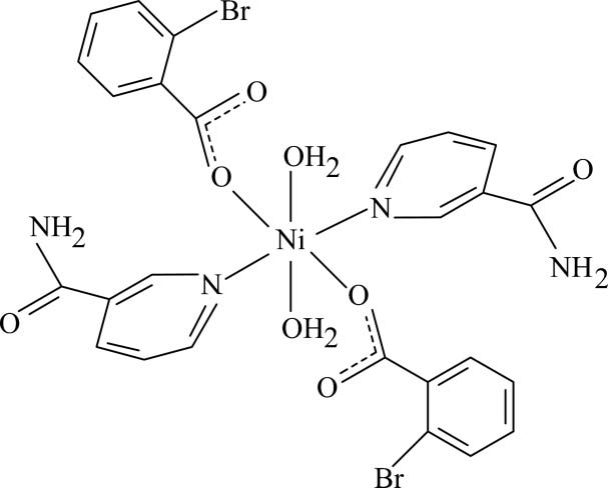


## Experimental

### 

#### Crystal data

[Ni(C_7_H_4_BrO_2_)_2_(C_6_H_6_N_2_O)_2_(H_2_O)_2_]
*M* *_r_* = 739.02
Monoclinic, 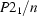

*a* = 7.8851 (2) Å
*b* = 18.2865 (4) Å
*c* = 9.7574 (3) Å
β = 106.609 (2)°
*V* = 1348.23 (6) Å^3^
*Z* = 2
Mo *K*α radiation
μ = 3.74 mm^−1^
*T* = 100 K
0.52 × 0.27 × 0.22 mm

#### Data collection

Bruker Kappa APEXII CCD area-detector diffractometer
Absorption correction: multi-scan (*SADABS*; Bruker, 2005 ▶) *T*_min_ = 0.306, *T*_max_ = 0.436
12686 measured reflections
3368 independent reflections
2899 reflections with *I* > 2σ(*I*)
*R*_int_ = 0.029

#### Refinement

*R*[*F* ^2^ > 2σ(*F* ^2^)] = 0.034
*wR*(*F* ^2^) = 0.090
*S* = 1.06
3368 reflections
203 parameters
H atoms treated by a mixture of independent and constrained refinement
Δρ_max_ = 1.48 e Å^−3^
Δρ_min_ = −0.55 e Å^−3^

### 

Data collection: *APEX2* (Bruker, 2007 ▶); cell refinement: *SAINT* (Bruker, 2007 ▶); data reduction: *SAINT*; program(s) used to solve structure: *SHELXS97* (Sheldrick, 2008 ▶); program(s) used to refine structure: *SHELXL97* (Sheldrick, 2008 ▶); molecular graphics: *ORTEP-3 for Windows* (Farrugia, 1997 ▶); software used to prepare material for publication: *WinGX* (Farrugia, 1999 ▶).

## Supplementary Material

Crystal structure: contains datablocks I, global. DOI: 10.1107/S1600536809021710/xu2538sup1.cif

Structure factors: contains datablocks I. DOI: 10.1107/S1600536809021710/xu2538Isup2.hkl

Additional supplementary materials:  crystallographic information; 3D view; checkCIF report

## Figures and Tables

**Table 1:** Selected bond lengths (Å)

Ni1—O1	2.0806 (16)
Ni1—O4	2.1012 (17)
Ni1—N1	2.068 (2)

**Table 2:** Hydrogen-bond geometry (Å, °)

*D*—H⋯*A*	*D*—H	H⋯*A*	*D*⋯*A*	*D*—H⋯*A*
N2—H21⋯O2^i^	0.82 (4)	2.09 (4)	2.864 (3)	156 (4)
N2—H22⋯O3^ii^	0.79 (4)	2.22 (4)	2.951 (3)	153 (4)
O4—H41⋯O2^iii^	0.86 (4)	1.77 (4)	2.612 (2)	165 (5)
O4—H42⋯O3^iv^	0.83 (4)	2.09 (4)	2.886 (2)	162 (3)
C9—H9⋯*Cg*1^v^	0.93	2.89	3.617 (3)	136

Symmetry codes: (i) 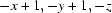; (ii) 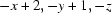; (iii) 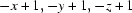; (iv) 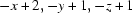; (v) 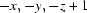. *Cg*1 is the centroid of the C2–C7 ring.

**Fig. 1.**
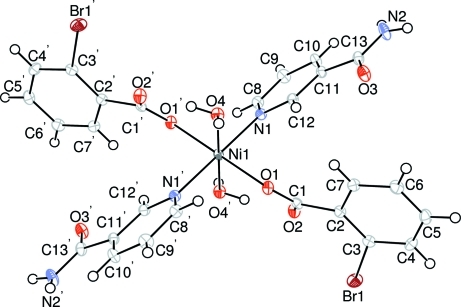
The molecular structure of the title molecule with the atom-numbering scheme. Displacement ellipsoids are drawn at the 50% probability level. Primed atoms are generated by the symmetry operator (1 - x, 1-y, 1-z).

## supplementary crystallographic information

### Comment

Transition metal complexes with biochemically active ligands frequently show interesting physical and/or chemical properties, as a result they may find applications in biological systems (Antolini *et al.*, 1982). The structural functions and coordination relationships of the arylcarboxylate ion in transition metal complexes of benzoic acid derivatives change depending on the nature and position of the substituent groups on the benzene ring, the nature of the additional ligand molecule or solvent, and the medium of the synthesis (Nadzhafov *et al.*, 1981; Shnulin *et al.*, 1981). Nicotinamide (NA) is one form of niacin and a deficiency of this vitamin leads to loss of copper from the body, known as pellagra disease. Victims of pellagra show unusually high serum and urinary copper levels (Krishnamachari, 1974). On the other hand, the nicotinic acid derivative *N*,*N*-diethylnicotinamide (DENA) is an important respiratory stimulant (Bigoli *et al.*, 1972).

The structure determination of the title compound, (I), a nickel complex with two 2-bromobenzoate (BB), two nicotinamide (NA) ligands and two water molecules, was undertaken in order to determine the properties of the ligands and also to compare the results obtained with those reported previously.

Compound (I) is a monomeric complex, with the Ni atom on a centre of symmetry. It contains two BB, two NA ligands and two water molecules (Fig. 1). All ligands are monodentate. The four O atoms (O1, O4, and the symmetry-related atoms, O1', O4') in the equatorial plane around the Ni atom form a slightly distorted square-planar arrangement, while the slightly distorted octahedral coordination is completed by the two N atoms of the NA ligands (N1, N1') in the axial positions (Table 1 and Fig. 1).

The near equality of the C1—O1 [1.260 (3) Å] and C1—O2 [1.258 (3) Å] bonds in the carboxylate group indicates a delocalized bonding arrangement, rather than localized single and double bonds, and may be compared with the corresponding distances: 1.262 (3) and 1.249 (3) Å in [Mn(DENA)_2_(C_8_H_5_O_3_)_2_(H_2_O)_2_], (II) (Sertçelik *et al.*, 2009*a*), 1.263 (4) and 1.249 (4) Å in [Ni(DENA)_2_(C_8_H_5_O_3_)_2_(H_2_O)_2_], (III) (Sertçelik *et al.*, 2009*b*), 1.262 (5) and 1.257 (5) Å in [Co(DENA)_2_(C_8_H_5_O_3_)_2_(H_2_O)_2_], (IV) (Sertçelik *et al.*, 2009*c*), 1.244 (4) and 1.270 (4) Å in [Co(NA)_2_(H_2_O)_4_](C_7_H_4_FO_2_)_2_, (V) (Özbek *et al.*, 2009), 1.284 (2), 1.248 (2) and 1.278 (2), 1.241 (2) Å in [Zn(NA)_2_(C_8_H_8_NO_2_)_2_], (VI) (Tercan *et al.*, 2009), 1.267 (3) and 1.258 (3) Å in [Ni(NA)_2_(C_7_H_4_ClO_2_)_2_(H_2_O)_2_], (VII) (Hökelek *et al.*, 2009*a*), 1.263 (2) and 1.240 (2) Å in [Zn(DENA)_2_(C_7_H_4_BrO_2_)_2_(H_2_O)_2_], (VIII) (Hökelek *et al.*, 2009*b*), and 1.2611 (17) and 1.2396 (19) Å in [Mn(DENA)_2_(C_7_H_4_BrO_2_)_2_(H_2_O)_2_], (IX) (Hökelek *et al.*, 2009*c*). In (I), the average Ni—O bond length is 2.0909 (17) Å and the Ni atom is displaced out of the least-squares plane of the carboxylate group (O1/C1/O2) by -0.595 (1) Å. The dihedral angle between the planar carboxylate group and the benzene ring A (C2—C7) is 30.81 (17)°, while that between rings A and B (N1/C8—C12) is 84.66 (6)°.

In the crystal structure, intermolecular O—H···O and N—H···O hydrogen bonds (Table 2) link the molecules into a supramolecular structure, in which they may be effective in the stabilization of the structure. A weak C—H···π interaction (Table 2) is also found.

### Experimental

The title compound was prepared by the reaction of Ni(SO_4_).6(H_2_O) (1.31 g, 5 mmol) in H_2_O (20 ml) and NA (1.22 g, 10 mmol) in H_2_O (20 ml) with sodium 2-bromobenzoate (2.23 g, 10 mmol) in H_2_O (50 ml). The mixture was filtered and set aside to crystallize at ambient temperature for 2 d, giving blue single crystals.

### Refinement

H atoms of water molecule and NH_2_ group were located in difference Fourier maps and refined isotropically. The remaining H atoms were positioned geometrically with C—H = 0.93 Å, for aromatic H atoms and constrained to ride on their parent atoms, with *U*_iso_(H) = 1.2*U*_eq_(C).

### Crystal data

**Table:**

[Ni(C_7_H_4_BrO_2_)_2_(C_6_H_6_N_2_O)_2_(H_2_O)_2_]	*F*(000) = 740
*M**_r_* = 739.02	*D*_x_ = 1.820 Mg m^−^^3^
Monoclinic, *P*2_1_/*n*	Mo *K*α radiation, λ = 0.71073 Å
Hall symbol: -P 2yn	Cell parameters from 5380 reflections
*a* = 7.8851 (2) Å	θ = 2.5–28.4°
*b* = 18.2865 (4) Å	µ = 3.74 mm^−^^1^
*c* = 9.7574 (3) Å	*T* = 100 K
β = 106.609 (2)°	Block, blue
*V* = 1348.23 (6) Å^3^	0.52 × 0.27 × 0.22 mm
*Z* = 2	

### Data collection

**Table:**

Bruker Kappa APEXII CCD area-detector diffractometer	3368 independent reflections
Radiation source: fine-focus sealed tube	2899 reflections with *I* > 2σ(*I*)
graphite	*R*_int_ = 0.029
φ and ω scans	θ_max_ = 28.4°, θ_min_ = 2.2°
Absorption correction: multi-scan (*SADABS*; Bruker, 2005)	*h* = −10→10
*T*_min_ = 0.306, *T*_max_ = 0.436	*k* = −24→21
12686 measured reflections	*l* = −12→13

### Refinement

**Table:**

Refinement on *F*^2^	Primary atom site location: structure-invariant direct methods
Least-squares matrix: full	Secondary atom site location: difference Fourier map
*R*[*F*^2^ > 2σ(*F*^2^)] = 0.034	Hydrogen site location: inferred from neighbouring sites
*wR*(*F*^2^) = 0.090	H atoms treated by a mixture of independent and constrained refinement
*S* = 1.06	*w* = 1/[σ^2^(*F*_o_^2^) + (0.0538*P*)^2^ + 0.96*P*] where *P* = (*F*_o_^2^ + 2*F*_c_^2^)/3
3368 reflections	(Δ/σ)_max_ < 0.001
203 parameters	Δρ_max_ = 1.48 e Å^−^^3^
0 restraints	Δρ_min_ = −0.55 e Å^−^^3^

### Special details

**Table:**

Geometry. All e.s.d.'s (except the e.s.d. in the dihedral angle between two l.s. planes) are estimated using the full covariance matrix. The cell e.s.d.'s are taken into account individually in the estimation of e.s.d.'s in distances, angles and torsion angles; correlations between e.s.d.'s in cell parameters are only used when they are defined by crystal symmetry. An approximate (isotropic) treatment of cell e.s.d.'s is used for estimating e.s.d.'s involving l.s. planes.
Refinement. Refinement of *F*^2^ against ALL reflections. The weighted *R*-factor *wR* and goodness of fit *S* are based on *F*^2^, conventional *R*-factors *R* are based on *F*, with *F* set to zero for negative *F*^2^. The threshold expression of *F*^2^ > σ(*F*^2^) is used only for calculating *R*-factors(gt) *etc*. and is not relevant to the choice of reflections for refinement. *R*-factors based on *F*^2^ are statistically about twice as large as those based on *F*, and *R*- factors based on ALL data will be even larger.

### Fractional atomic coordinates and isotropic or equivalent isotropic displacement parameters (Å^2^)

**Table:**

	*x*	*y*	*z*	*U*_iso_*/*U*_eq_	
Br1	0.40260 (3)	0.212024 (14)	0.17902 (3)	0.02187 (10)	
Ni1	0.5000	0.5000	0.5000	0.01048 (11)	
O1	0.6201 (2)	0.40537 (9)	0.45455 (18)	0.0137 (3)	
O2	0.3946 (2)	0.36790 (10)	0.27275 (19)	0.0162 (4)	
O3	0.9542 (2)	0.51033 (10)	0.17931 (19)	0.0184 (4)	
O4	0.7554 (2)	0.54252 (10)	0.58888 (19)	0.0149 (4)	
H41	0.724 (6)	0.574 (2)	0.643 (4)	0.045 (11)*	
H42	0.838 (5)	0.5193 (19)	0.643 (4)	0.024 (8)*	
N1	0.5076 (3)	0.54372 (11)	0.3063 (2)	0.0129 (4)	
N2	0.8578 (3)	0.57987 (14)	−0.0186 (2)	0.0194 (5)	
H21	0.776 (5)	0.6019 (19)	−0.074 (4)	0.034 (10)*	
H22	0.935 (5)	0.5647 (18)	−0.048 (4)	0.023 (8)*	
C1	0.5546 (3)	0.36682 (13)	0.3452 (2)	0.0126 (4)	
C2	0.6793 (3)	0.31957 (13)	0.2910 (3)	0.0130 (4)	
C3	0.6274 (3)	0.25649 (14)	0.2082 (3)	0.0138 (5)	
C4	0.7416 (3)	0.22059 (13)	0.1454 (3)	0.0160 (5)	
H4	0.7036	0.1793	0.0893	0.019*	
C5	0.9119 (3)	0.24655 (15)	0.1666 (3)	0.0176 (5)	
H5	0.9876	0.2235	0.1227	0.021*	
C6	0.9699 (3)	0.30671 (14)	0.2531 (3)	0.0175 (5)	
H6	1.0859	0.3230	0.2705	0.021*	
C7	0.8537 (3)	0.34266 (13)	0.3137 (3)	0.0143 (5)	
H7	0.8934	0.3833	0.3711	0.017*	
C8	0.3703 (3)	0.58033 (13)	0.2194 (3)	0.0143 (5)	
H8	0.2668	0.5848	0.2464	0.017*	
C9	0.3762 (3)	0.61133 (14)	0.0923 (3)	0.0167 (5)	
H9	0.2792	0.6367	0.0354	0.020*	
C10	0.5299 (3)	0.60410 (14)	0.0504 (3)	0.0159 (5)	
H10	0.5376	0.6246	−0.0348	0.019*	
C11	0.6709 (3)	0.56580 (13)	0.1378 (3)	0.0131 (5)	
C12	0.6545 (3)	0.53690 (13)	0.2651 (3)	0.0134 (5)	
H12	0.7500	0.5117	0.3243	0.016*	
C13	0.8401 (3)	0.55041 (14)	0.1008 (3)	0.0152 (5)	

### Atomic displacement parameters (Å^2^)

**Table:**

	*U*^11^	*U*^22^	*U*^33^	*U*^12^	*U*^13^	*U*^23^
Br1	0.01372 (14)	0.01967 (16)	0.03198 (17)	−0.00559 (9)	0.00611 (11)	−0.00455 (10)
Ni1	0.00719 (19)	0.0141 (2)	0.0102 (2)	0.00053 (14)	0.00254 (15)	0.00033 (15)
O1	0.0115 (8)	0.0168 (9)	0.0122 (8)	0.0021 (6)	0.0022 (6)	−0.0009 (6)
O2	0.0082 (7)	0.0226 (10)	0.0164 (8)	0.0018 (6)	0.0015 (6)	−0.0023 (7)
O3	0.0133 (8)	0.0269 (10)	0.0156 (9)	0.0064 (7)	0.0049 (7)	0.0048 (7)
O4	0.0083 (8)	0.0191 (10)	0.0166 (9)	0.0012 (7)	0.0024 (7)	−0.0005 (7)
N1	0.0108 (9)	0.0138 (10)	0.0142 (10)	0.0004 (7)	0.0036 (8)	−0.0001 (8)
N2	0.0131 (10)	0.0324 (13)	0.0143 (10)	0.0074 (9)	0.0063 (9)	0.0062 (9)
C1	0.0105 (10)	0.0152 (12)	0.0126 (11)	0.0013 (8)	0.0043 (9)	0.0026 (9)
C2	0.0103 (10)	0.0143 (12)	0.0143 (11)	0.0018 (8)	0.0034 (9)	0.0015 (9)
C3	0.0102 (10)	0.0162 (12)	0.0137 (11)	−0.0005 (8)	0.0013 (9)	0.0018 (9)
C4	0.0166 (11)	0.0127 (12)	0.0172 (12)	0.0027 (9)	0.0025 (9)	−0.0012 (9)
C5	0.0165 (12)	0.0207 (14)	0.0166 (12)	0.0065 (9)	0.0064 (10)	0.0025 (10)
C6	0.0113 (11)	0.0205 (13)	0.0209 (12)	−0.0004 (9)	0.0051 (10)	0.0010 (10)
C7	0.0110 (10)	0.0149 (12)	0.0165 (11)	0.0010 (8)	0.0031 (9)	0.0005 (9)
C8	0.0095 (10)	0.0163 (12)	0.0167 (11)	0.0020 (8)	0.0029 (9)	0.0001 (9)
C9	0.0093 (10)	0.0211 (13)	0.0175 (12)	0.0051 (9)	0.0002 (9)	0.0043 (10)
C10	0.0132 (11)	0.0224 (13)	0.0130 (11)	0.0022 (9)	0.0052 (9)	0.0032 (9)
C11	0.0117 (11)	0.0146 (12)	0.0139 (11)	0.0007 (8)	0.0051 (9)	−0.0004 (9)
C12	0.0117 (10)	0.0135 (12)	0.0155 (11)	0.0011 (8)	0.0046 (9)	0.0000 (9)
C13	0.0113 (11)	0.0203 (13)	0.0145 (11)	0.0005 (9)	0.0044 (9)	−0.0019 (9)

### Geometric parameters (Å, °)

**Table:**

Br1—C3	1.896 (2)	C3—C4	1.390 (3)
Ni1—O1^i^	2.0806 (16)	C4—C5	1.383 (4)
Ni1—O1	2.0806 (16)	C4—H4	0.9300
Ni1—O4	2.1012 (17)	C5—C6	1.382 (4)
Ni1—O4^i^	2.1012 (17)	C5—H5	0.9300
Ni1—N1	2.068 (2)	C6—C7	1.390 (3)
Ni1—N1^i^	2.068 (2)	C6—H6	0.9300
O1—C1	1.260 (3)	C7—H7	0.9300
O2—C1	1.258 (3)	C8—H8	0.9300
O3—C13	1.241 (3)	C9—C8	1.377 (3)
O4—H41	0.86 (4)	C9—H9	0.9300
O4—H42	0.83 (4)	C10—C9	1.391 (3)
N1—C8	1.347 (3)	C10—H10	0.9300
N1—C12	1.337 (3)	C11—C10	1.383 (3)
N2—H21	0.82 (4)	C11—C12	1.390 (3)
N2—H22	0.79 (4)	C12—H12	0.9300
C1—C2	1.513 (3)	C13—N2	1.327 (3)
C2—C7	1.394 (3)	C13—C11	1.505 (3)
C3—C2	1.400 (4)		
			
O1^i^—Ni1—O1	180.000 (1)	C4—C3—Br1	115.24 (18)
O1—Ni1—O4	87.40 (7)	C4—C3—C2	121.6 (2)
O1^i^—Ni1—O4	92.60 (7)	C3—C4—H4	120.2
O1^i^—Ni1—O4^i^	87.40 (7)	C5—C4—C3	119.7 (2)
O1—Ni1—O4^i^	92.60 (7)	C5—C4—H4	120.2
O4—Ni1—O4^i^	180.00 (10)	C4—C5—H5	119.9
N1—Ni1—O1	89.60 (7)	C6—C5—C4	120.2 (2)
N1^i^—Ni1—O1	90.40 (7)	C6—C5—H5	119.9
N1—Ni1—O1^i^	90.40 (7)	C5—C6—C7	119.5 (2)
N1^i^—Ni1—O1^i^	89.60 (7)	C5—C6—H6	120.3
N1—Ni1—O4	87.68 (7)	C7—C6—H6	120.3
N1^i^—Ni1—O4	92.32 (7)	C2—C7—H7	119.0
N1—Ni1—O4^i^	92.32 (7)	C6—C7—C2	122.0 (2)
N1^i^—Ni1—O4^i^	87.68 (7)	C6—C7—H7	119.0
N1—Ni1—N1^i^	180.00 (10)	N1—C8—C9	123.0 (2)
C1—O1—Ni1	122.99 (15)	N1—C8—H8	118.5
Ni1—O4—H41	95 (3)	C9—C8—H8	118.5
Ni1—O4—H42	124 (2)	C8—C9—C10	118.8 (2)
H42—O4—H41	105 (4)	C8—C9—H9	120.6
C8—N1—Ni1	122.66 (15)	C10—C9—H9	120.6
C12—N1—Ni1	119.53 (16)	C9—C10—H10	120.6
C12—N1—C8	117.8 (2)	C11—C10—C9	118.8 (2)
C13—N2—H21	121 (3)	C11—C10—H10	120.6
C13—N2—H22	118 (2)	C10—C11—C12	118.7 (2)
H21—N2—H22	118 (3)	C10—C11—C13	124.0 (2)
O1—C1—C2	117.8 (2)	C12—C11—C13	117.3 (2)
O2—C1—O1	124.7 (2)	N1—C12—C11	123.0 (2)
O2—C1—C2	117.4 (2)	N1—C12—H12	118.5
C3—C2—C1	124.1 (2)	C11—C12—H12	118.5
C7—C2—C1	118.8 (2)	O3—C13—N2	122.8 (2)
C7—C2—C3	117.0 (2)	O3—C13—C11	119.9 (2)
C2—C3—Br1	123.11 (17)	N2—C13—C11	117.2 (2)
			
O4—Ni1—O1—C1	−145.89 (18)	C1—C2—C7—C6	−172.5 (2)
O4^i^—Ni1—O1—C1	34.11 (18)	C3—C2—C7—C6	2.3 (4)
N1—Ni1—O1—C1	−58.20 (18)	Br1—C3—C2—C1	−10.9 (3)
N1^i^—Ni1—O1—C1	121.80 (18)	Br1—C3—C2—C7	174.53 (17)
O1—Ni1—N1—C8	137.00 (19)	C4—C3—C2—C1	171.3 (2)
O1^i^—Ni1—N1—C8	−43.00 (19)	C4—C3—C2—C7	−3.2 (4)
O1—Ni1—N1—C12	−44.08 (18)	Br1—C3—C4—C5	−176.67 (19)
O1^i^—Ni1—N1—C12	135.92 (18)	C2—C3—C4—C5	1.2 (4)
O4—Ni1—N1—C12	43.33 (18)	C3—C4—C5—C6	1.7 (4)
O4^i^—Ni1—N1—C12	−136.67 (18)	C4—C5—C6—C7	−2.6 (4)
O4—Ni1—N1—C8	−135.59 (19)	C5—C6—C7—C2	0.5 (4)
O4^i^—Ni1—N1—C8	44.41 (19)	C10—C9—C8—N1	0.7 (4)
Ni1—O1—C1—O2	−19.9 (3)	C11—C10—C9—C8	0.2 (4)
Ni1—O1—C1—C2	155.74 (16)	C12—C11—C10—C9	−0.9 (4)
Ni1—N1—C8—C9	178.11 (19)	C13—C11—C10—C9	176.4 (2)
C12—N1—C8—C9	−0.8 (4)	C10—C11—C12—N1	0.8 (4)
Ni1—N1—C12—C11	−178.89 (18)	C13—C11—C12—N1	−176.7 (2)
C8—N1—C12—C11	0.1 (4)	O3—C13—C11—C10	−173.8 (2)
O1—C1—C2—C3	156.0 (2)	O3—C13—C11—C12	3.5 (4)
O1—C1—C2—C7	−29.6 (3)	N2—C13—C11—C10	4.7 (4)
O2—C1—C2—C3	−28.0 (4)	N2—C13—C11—C12	−178.0 (2)
O2—C1—C2—C7	146.4 (2)		

Symmetry codes: (i) −*x*+1, −*y*+1, −*z*+1.

### Hydrogen-bond geometry (Å, °)

**Table:**

*D*—H···*A*	*D*—H	H···*A*	*D*···*A*	*D*—H···*A*
N2—H21···O2^ii^	0.82 (4)	2.09 (4)	2.864 (3)	156 (4)
N2—H22···O3^iii^	0.79 (4)	2.22 (4)	2.951 (3)	153 (4)
O4—H41···O2^i^	0.86 (4)	1.77 (4)	2.612 (2)	165 (5)
O4—H42···O3^iv^	0.83 (4)	2.09 (4)	2.886 (2)	162 (3)
C9—H9···Cg1^v^	0.93	2.89	3.617 (3)	136

Symmetry codes: (ii) −*x*+1, −*y*+1, −*z*; (iii) −*x*+2, −*y*+1, −*z*; (i) −*x*+1, −*y*+1, −*z*+1; (iv) −*x*+2, −*y*+1, −*z*+1; (v) −*x*, −*y*, −*z*+1.
